# Follow-Up in Aphasia Caused by Acute Stroke in a Prospective, Randomized, Clinical, and Experimental Controlled Noninvasive Study With an iPad-Based App (Neolexon®): Study Protocol of the Lexi Study

**DOI:** 10.3389/fneur.2020.00294

**Published:** 2020-04-30

**Authors:** Dennis C. Thunstedt, Peter Young, Clemens Küpper, Katharina Müller, Regina Becker, Franziska Erbert, Katharina Lehner, Marika Rheinwald, Angelika Pfahler, Marianne Dieterich, Lars Kellert, Katharina Feil

**Affiliations:** ^1^Department of Neurology, Ludwig Maximilians University (LMU), Munich, Germany; ^2^Department of Neurology, Medical Park Bad Feilnbach, Reithofpark, Germany; ^3^Clinic for Orthopedic Surgery, Physical Medicine and Rehabilitation, Munich, Germany; ^4^German Center for Vertigo and Balance Disorders, Ludwig Maximilians University (LMU), Munich, Germany; ^5^Munich Cluster for Systems Neurology (SyNergy), Munich, Germany

**Keywords:** acute aphasia, ischemic stroke, hemorrhagic stroke, acute stroke, tablet-based therapy, speech therapy, app, Neolexon®

## Abstract

**Rationale:** Treatment of aphasia is still challenging for clinicians and patients. So far, there is proven evidence for “face-to-face” speech therapy. However, the digital age potentially offers new and complementary strategies that may add to treatment outcome in a cost-effective way. Neolexon® is a commercial tablet-based software for treatment of aphasia, which can be applied with the help of a therapist or as self-training by the patient.

**Aims and hypothesis:** In the Lexi study, we aim to determine whether treatment with Neolexon® is superior to standard therapy in acute post-stroke aphasia.

**Sample size estimates:** A sample size of 180 patients, 90 for each group, will be included with an assumed dropout rate of ~20%.

**Methods and design:** Prospective, randomized, parallel group, open-label, blinded-endpoint clinical, and experimental controlled non-invasive trial (PROBE). Adult German native speakers with acute aphasia after stroke are included. Computer-generated, blocked, and stratified randomization by aphasia severity will assign patients to one of two groups: 4 weeks of either standard logopedic speech therapy or logopedic speech therapy with the app version of Neolexon®. Both groups will be instructed in self-training: the frequency and duration of self-training will be documented. Screening for aphasia will be performed using the Language Screening Test (LAST). The severity of aphasia in general and in subitems will be assessed using the Bielefelder Aphasie Screening (BIAS) and the Aphasia Check List (ACL). Follow-up will be assessed after 3 months.

**Study outcomes:** Based on the consensus in our study team, we considered a 10% mean difference in the change of percentile rank (PR) of BIAS to be a minimal and clinically important difference. The primary endpoint is defined as a significant difference in BIAS comparing the two groups. Differences in quality of life, Beck Depression Inventory (BDI), and modified Ranking Scale (mRS) will be evaluated as secondary outcome parameters.

**Discussion:** This trial will determine whether speech therapy with the use of Neolexon® is superior to standard logopedic therapy. Subgroups with the greatest response to Neolexon® will be described. The trial was prospectively registered on the “EU Clinical Trials Register” (NCT04080817)^1^.

## Introduction

Aphasia as a neuropsychological language disability is a condition affecting communication, cognition, and identity negatively (1). Therefore, aphasic syndromes are connected to affective disorders like depression (2) or fatigue and are associated with a negative impact on quality of life. The latter is particularly important with regard to the advantages of the self-therapy (3–5). The incidence of aphasia is 43/100,000, and it is a first and leading symptom in a significant proportion of stroke patients (6, 7). In Germany, it is assumed that about 70,000 people suffer from aphasia (8). Aphasic syndromes are frequently seen in stroke caused by lesions of the dominant hemisphere (3); however, they can also be caused by trauma, epilepsy, or degenerative diseases (9). The effect of “standard” speech therapy in patients with aphasia is already scientifically proven (10–12). Regarding the therapy of aphasia, intensified logopedic standard therapy more than 5 h per week was shown to be effective (10, 13). Moreover, intensive speech therapy for 3 weeks (more than 10 h/week) was suggested to improve communication skills significantly (11). In addition, communication-based treatment as well as logopedic group therapy were shown to have a positive effect as well (13, 14) Early treatment and high intensity are potentially effective (13), especially in stroke patients (15), but it should be noted that not every high-intensity therapy is applicable to all patients. Further, results regarding very early rehabilitation after stroke are lacking (15). The German Neurology Society, therefore, underlines the urgency of immediate treatment. Furthermore, 5–10 h per week should be applied to show a significant benefit (16). However, it can be difficult to meet these demands in clinical practice.

Software-based treatment and the use of modern devices can offer a potential and modern addendum to face-to-face speech-language therapy of aphasic patients. In the digital age and its applications on tablets and smartphones, it is important to investigate modern treatment options especially in the neurologic field. A small population study investigating the effect of computer-assisted self-training vs. nonlinguistic cognitive therapy in 18 patients showed improved communication skills (17, 18). Due to different limitations concerning the population, further investigations are necessary in the future (19). A fundamental study in this field was the “BIG CACTUS” trial: 240 patients with post-stroke aphasia improved in word finding after computer-based treatment, although there was no enhancement seen in conversation skills. In this trial, both groups with post-stroke aphasia received the usual care, but the study group was additionally treated with computerized self-therapy. However, this trial was a power analysis regarding chronic aphasia (20). Another notable study researching the outcome of electrical brain stimulation on post-stroke aphasia demonstrated effectiveness in 26 patients (21).

Neolexon® is a tablet-based application offering tailored training lessons for patients with aphasia. Neolexon® was developed by two speech therapists Mona Späth and Hanna Jakob and programmed by IT experts from the Ludwig Maximilians University (LMU) Munich. It is certified as a health care product^2^ In this study, we seek to determine whether a standard logopedic therapy is less effective than a tablet-based speech therapy with Neolexon® in post-stroke aphasic patients. The aim is to prove the effectiveness of digital applications on aphasia after (ischemic or hemorrhagic) stroke.

## Methods and Analysis

### Design

The Lexi Study is a prospective, randomized, open-label, clinical, and experimental controlled noninvasive trial with two groups. Clinical follow-up will be obtained during an outpatient clinic visit within an endpoint-blinded design. The trial was approved by the ethics committee of the LMU, Munich, Germany (project number 19-068). The trial was prospectively registered on the “EU Clinical Trials Register” (NCT04080817)^1^.

The Neolexon® App is an individualized software that can be used together with a speech therapist (therapist app version) or alone (self-training app) at home. Any user can compile training sessions from up to 8,000 words and 1,200 sentences and sort them into different categories. Pictures, which are also used in standard speech therapy are also digitally established in Neolexon®. The speech therapist generates an individual profile of the patient and can adjust the number of syllables, syllable structure, word accent, and word frequency. Words can then be searched by category, for example, “food,” and pictures can be displayed that the patient has to name. Neolexon® is intended for auditory speech comprehension, oral and written naming, and reading comprehension training. During self-training, the patient can then log in by clicking on the “patient” button and continue training with his adjusted profile without restriction. The application detects therapy success automatically and offers continuous adjustment during the exercise. In addition, the application systematically records the duration of all training lessons^3^

### Selection/Treatment of Subjects

Patients fulfilling the inclusion criteria at screening will be randomized in a ratio of 1:1 into two comparative treatment groups. Patients not capable of giving written informed consent due to physical or mental disorders can be included in the study after written informed consent of the legal guardian. On the other hand, if the inability to give permission is transient, the patient has to acquiesce afterwards.

The procedure considers stratification by aphasia severity to ensure balanced strata, maintaining allocation concealment. The Medical Informatics, Biometry, and Epidemiology (IBE) of the University of Munich will provide an internet-based, password-protected randomization tool “Randoulette^4^,” which chooses the treatment sequence for a new patient who fulfills the eligibility criteria and has signed the informed consent. Randoulette will register the patient by his or her screening number, gender, year of birth, and strata before the allocated number is provided.

Patients will be screened directly after admission to hospital for stroke. The study population includes a broad range of patients with acute aphasia after any stroke (ischemic or hemorrhagic). Inclusion and exclusion criteria are shown in Table 1.

**Table 1:** Inclusion and exclusion criteria for Lexi study.

**Inclusion criteria**
≥18 years of age
German mother tongue
Acute aphasia after stroke
Life expectancy > 1 year
Written informed consent
**Exclusion criteria**
<18 years of age
Non-native German speaker
Other causes of aphasia
Life expectancy <1 year
Informed consent missing
Impossible to use tablet-based app due to physical or mental conditions

### Interventional Methods

Patients will be screened and assessed for eligibility directly after admission to our stroke unit. Patients eligible for entry to the study will be randomized and assigned to one of the two treatment arms after written informed consent. Each group (standard speech therapy vs. speech therapy with the therapist app version of Neolexon® therapy) should have a minimum of 50 participants. Because of the dropout rate of ~20%, at least 70 patients should be included in each group.

**Figure 1:**
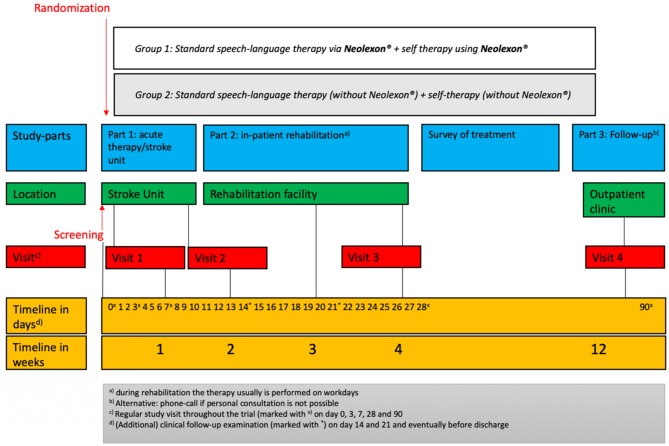
Timeline of this study.

Each study arm (treatment group and control group) with a duration of 4 weeks includes three study visits. Follow-up will be assessed after 3 months (study visit 4). Figure 1 shows the study scheme and Table 2 demonstrates the schedule of enrollment and assessment as well as interventions at each study visit during the Lexi study.

**Table 2:** Schedule of enrollment, interventions, and assessments.

	**Part 1**			**Part 2**	**Part 3**
	**Enrollment**	**Visit 1**	**Visit 2**	**Visit 3**	**Visit 4^a^)**
Timeline	Diagnosis of stroke	During hospital stay	Before rehabilitation	End of intervention	Follow-up
	Admission				
	Day 0 + 3 days	Day 3 + 3	Day 7 ± 3	Day 28 ± 3	3 months ± 7 days
Check for inclusion/exclusion criteria	X				
Randomization	X				
Informed consent^b^	X				
Baseline data	X				
Neurological assessment	X		X	X	X
Edinburgh Handedness Inventory (EHI)	X				
(p)mRS	X		X	X	X
NIHSS	X	X	X	X	X
BI	X		X	X	X
LAST	X	X	X	X	X
ACL	X	X	X	X	X
BIAS	X	X	X	X	X
BDI	X		X	X	X
(EQ-5D-5L)	X		X	X	X
Evaluation of self-training (h/day)		X	X	X	X

a *Alternative: phone call if personal consultation is not possible*.

b *If not able to get permission from the patients themselves, their legal guardian will have to give written informed consent for their contribution*.

*ACL, Aphasia Check List; BDI, Beck Depression Inventory; BIAS, Bielefelder Aphasie Screening; BI, Barthel Index; CNS, central nervous system; mRS, modified Rankin-Scale; NIHSS, National Institutes of Health Stroke Scale; LAST, Language Screening Test*.

The group with Neolexon® will receive logopedic therapy with the help of Neolexon® (exercise programs for speech therapists in the program, therapist app version). The control group will receive standard speech therapy without Neolexon®. Both groups will be instructed in self-training in the control group without the help of the app and in the treatment group with Neolexon®. In both groups, the frequency, duration, and type of self-training will be documented. The total study duration is 12 weeks and is divided into three parts:

- Part 1 consists of screening and enrollment in the study. Part 1 will take place in our facility at the acute phase of ischemic or hemorrhagic stroke for ~1 week and includes two study visits: visit 1 and visit 2. During study visit 1, initial baseline information, including demographic, epidemiologic, and clinical data as well as comorbidities, are obtained, including neurological assessment using various screening tools at inclusion, aphasia rating scales as well as patient questionnaires for quality of life and depression. It is important to note that clinical examinations will take place outside the regular study visits at day 14 and 21 and potentially before a change of location.
- Part 2 of our trial concludes after discharge to a rehabilitation facility. Visit 3 will be performed in this context. On this occasion, the abovementioned tests and psychological questionnaires from the enrollment are repeated. Visit 3 will take place ~4 weeks after screening as a follow-up visit.
- Between visit 3 and visit 4, the intensity of the intervention (self-exercises, speech therapy) is assessed, but there is no longer any need to comply with randomization.

Part 3 of the study includes the follow-up after 3 months: Clinical follow-up will be obtained during an outpatient clinic visit as an endpoint-blinded design, If this is not possible, telephone follow-up will be assessed.

As for the neurological examination, information including etiology of stroke or specific therapy regimens is collected. The severity of the stroke will be measured throughout using the (premorbid) modified Ranking-Scale (pmRS/mRS) (22), the National Health Institute of Stroke Scale (NIHSS) (23), as well as the Barthel Index (BI) as a well-known instrument describing independence for daily life skills. Scoring 100 means fully capable of self-care whereas 0 implies the opposite (24).

Screening for aphasia will be performed using the Language Screening Test (LAST) (25, 26). The LAST is a simple, quick, validated screening instrument focusing on speech comprehension, word repetition, and naming. LAST is conducted using the following parts: “Naming,” “Repetition,” “Automatic speech,” “Picture recognition,” and “Verbal instructions.” A total score of 15 can be achieved, meaning one point per item. The minimum number of points that can be attained is 0 (25, 26). In clinical trials, there is no validation of severity of aphasia using the LAST; however, based on clinical experience and consensus, the severity of aphasia is as follows: 0–5 severe, 6–10 moderate, and 11–14 minimal.

The Aphasia Check List (ACL), developed by Kalbe et al. (27) comprises two main parts: The first part, “Language,” covers a broad range of linguistic aspects, i.e., speaking in series or reading out loud. Communication abilities are evaluated on the basis of a four-level rating scale: From 0 meaning “severe aphasia” to 3 implying lack of disability. Also, writing after dictation is one noteworthy subtest. The second part, “Cognition,” focuses on non-verbal recall using tasks that require logical thinking, attention, and memory. After adding up all subtests, a cutoff score at 135 is defined as aphasia. The ACL takes up to 30 min (27). The ACL is a test that actually allows the severity of aphasia to be quantified. It is known to have test–retest reliability, and it has been used in other studies. However, the ACL does not include all the important speech/language modalities, so the primary endpoint is determined using the Bielefelder Aphasie Screening (BIAS).

The primary endpoint of the study is examined using the BIAS to assess the severity and course of aphasia in general as well as in subitems (28). The BIAS is a diagnostic tool that can be administered in the acute phase within 20–40 min with relatively little effort in clinical routine depending on aphasia severity. The BIAS addresses all modalities of language, from reading comprehension to spontaneous speech. The latter is used at the beginning of the evaluation as a first assessment. Aphasia is judged based on the performance in the seven subscales: 1. Spontaneous speech, 2. Auditory comprehension, 3. Automatic language use, 4. Semantic lexical performance 5. Reading comprehension, 6. Writing of words. All subtests are documented on a protocol sheet and scores are added up at the end. Finally, the severity of aphasia is graded using a percentile rank (PR) from 100%, which refers to a healthy person, to 0% (28).

Furthermore, as depression and reduced quality of life are common comorbidities in patients with stroke, patient-reported outcomes using the EuroQol 5 dimensions and 5 level version (EQ-5D-5L) and Beck's Depression Inventory (BDI-II) will be recorded with the help of a trained speech-language therapist or neuropsychologist. The EQ-5D-5L is a self-administered questionnaire to evaluate the quality of life. It is a standardized measure of health status providing a simple, generic measure of health for clinical and economic appraisal and consisting of two parts—the EQ-5D descriptive system and the EQ visual analog scale (EQ-VAS). The EQ-5D-5L descriptive system comprises the following five dimensions: mobility, self-care, usual activities, pain/discomfort, and anxiety/depression. Each dimension has five levels: no problems, slight problems, moderate problems, severe problems, and extreme problems (29).

The BDI-II is a multiple-choice self-report inventory for measuring the severity of depression and is composed of items related to symptoms of depression such as hopelessness and irritability, emotions such as guilt or feelings of being punished, as well as physical symptoms such as fatigue, weight loss, and lack of interest in sex (30).

### Data Analysis

Results from the study assessments will be recorded in a paper-based case report form (CRF). This form includes all the abovementioned screening tools plus questionnaires, and the survey of the intensity and frequency of applied speech therapy as well as (self-)training lessons. It is intended that each anonymous CRF will be filled in by our staff (speech therapists, doctoral candidates, or physicians) and then transferred either directly or afterwards into a central database on our server, so that the responsible individuals have parallel access to the data to maintain them. The paper form is collected and stored closed at the same time at our facility.

#### Primary Objective

Based on the consensus in our study team, we considered a 10% mean difference in the change of the PR of BIAS to be a minimal and clinically important difference. Our primary objective is to demonstrate a significant improvement of 10% in BIAS comparing the two groups. Differences in quality of life, BDI, and mRS will we evaluated as secondary outcome parameters.

We aimed to recruit 180 participants (90 patients per group), which had 85% power for a 5% two-sided test to address the primary objective. Sample size was adjusted for an assumed dropout rate of ~20%; therefore, at least 144 patients (72 per group) should be analyzed. We expect about 50% of screen fails—based on the calculated sample size, we need to screen 360 patients in our stroke unit.

Data with normal distribution will be presented as mean ± standard deviation (SD), and non-normally distributed data will be presented as median and range (min, max) or interquartile range (IQR). For categorical variables, counts and percentages will be given. Data will be compared with the chi-square test, Mann–Whitney test, or Student's *t*-test where appropriate. A two-sided *p*-value of <0.05 will be considered statistically significant. Binary logistic regression models will be used to analyze the association between study group and outcome parameters. Results will be adjusted for well-known outcome predictors after stroke, e.g., age, sex, NIHSS, pmRS, lesion size, treatment, comorbidities, and complications. Statistical analysis will be performed with the Statistical Package for the Social Sciences, SPSS (SPSS Inc., 21.0 for Windows).

## Discussion

The Lexi study will clarify whether patients benefit from speech therapy using the new individualized tablet-based Neolexon® app compared to standard “face-to-face” speech therapy without such a modern device. Both groups will be instructed in self-training. The study will further clarify if using this modern tablet-based Neolexon® app leads to more motivation and therefore a higher amount of self-training. Aphasia is caused by ischemic or hemorrhagic strokes most probably on the left side of the brain (31). The prognosis of aphasic syndromes depends on the severity at the beginning of the aphasia and the intensity of the stroke, while age and sex seem unrelated to the outcome (32). Nevertheless, not only the severity of the aphasia, but also word repetition seems to contribute significantly to the prognosis of aphasia (33).

Intensive and early speech therapy is associated with a good outcome (34). Flöel et al. (35) note that it is the combination of several methods rather than an isolated computer-based therapy regime that should improve aphasia, because face-to-face communication cannot be replaced.

Due to the large number of cerebrovascular events nowadays, it is important to establish modern and new effective treatment options (36). Given the burden of stroke on global health and the expected demographic changes in many countries, this could open up new possibilities in rehabilitation of aphasic syndromes after stroke.

In terms of the financial aspect of stroke treatment, costs will rise in the future. In 2004, 7.1 billion euros were spent on ischemic stroke patients in Germany (37). Application-based therapy can reduce costs in the healthcare system. Another key factor is that application-based speech therapy can overcome time gaps during any transfer to another clinic.

Technology in treatment of patients after stroke in general is already used, for instance in motor dysfunction (38) or, regarding to aphasia, in the approach of augmented embodied therapy for showing evidence of effectiveness (39). Another noteworthy option in the treatment of aphasia is anodal transcranial direct stimulation, which could be effective (40). In terms of software, there is “Tactus Therapy,” which not only addresses aphasia therapy in general but also trains apraxia, dysphagia, and communication skills. The aphasia app has four sections: naming, comprehension, reading, and writing^5^ As far as speech therapy with modern devices is concerned, “Constant Therapy” must also be mentioned; it uses two main columns: language and cognition. Different subcategories, such as writing, naming, and reading, should be mentioned here (41).

Since intensity of training is an important factor, which is difficult to provide due to limited human and financial resources, alternative methods have to be found in which self-training without the need for a therapist—or with a therapist only required at the beginning of the therapy—have a positive impact on aphasia patients (42).

Digital aphasia therapy makes it easier to reach patients in rural areas and may also be applicable in those countries, in which language therapy or rehabilitation facilities are not available.

The aim of our trial is to predict the outcome of a new software-based speech therapy on post-stroke aphasia.

The key steps of this study are as follows:

1. To enroll a selected population of patients with aphasia after proven etiology of any kind of stroke and randomize them into two groups.
2. To apply Neolexon® in our cohort and compare it to the control group while treating both groups with language therapy. During therapy, different scoring systems, including ACL, LAST, and BIAS, will be evaluated.

One of the potential limitations of our work is that only patients with post-stroke aphasia are included. There will be no conclusive data from aphasia after other causes. In addition, we decided not to investigate chronic aphasia. However, we are considering the possibility of performing further trials on these topics. Non-fluent German speakers and severely affected patients who are unable to use the tablet-based app due to the fact that physical or mental conditions are excluded, as are patients with a reduced life expectancy. Due to the lack of previous studies, we cannot conduct a sample size calculation based on treatment effects. Thus, this study could be underpowered to show an effect in the interventional group.

Our goal is to show whether patients could benefit from Neolexon® computer-based speech therapy. Further studies might be necessary to approach the main problem of other aspects of aphasia. Other important factors that could be interesting to analyze are motivation for speech therapy, reducing negative affective symptoms during treatment, and lower workload for other family members (43).

## Ethics Statement

The trial was approved by the ethics committee of the Ludwig Maximilian University (LMU), Munich, Germany (project number 19-068). In all cases, patients have to give written informed consent or, if they are not able to give permission themselves, their legal guardian will have to give written informed consent for their contribution. The trial was registered at the EU Clinical Trials Register^6^ (NCT04080817, registered on 06/09/2019).

## Author Contributions

All authors have read and approved the manuscript before submission. DT drafting/revising of the manuscript for content, including medical writing. PY, CK, KM, RB, FE, KL, MR, AP and MD revising manuscript for content, including medical writing. LK and KF drafting/revising of the manuscript for content, including medical writing, study concept, design, interpretation of data, acquisition of data, development of clinical algorithm.

## Conflict of Interest

The study received funding from Boehringer Ingelheim Pharma GmbH & Co. KG and app licences Neolexon®. The authors declare that the research was conducted in the absence of any other commercial or financial relationships that could be construed as a potential conflict of interest.

## Footnotes

^1^www.clinicaltrialsregister.gov.

^2^https://neolexon.de.

^3^https://neolexon.de/patienten/aphasie-app/.

^4^https://wwwapp.ibe.med.uni-muenchen.de/randoulette/.

^5^https://tactustherapy.com.

^6^www.clinicaltrialsregister.eu.
